# Difference in quantity discrimination in dogs and wolves

**DOI:** 10.3389/fpsyg.2014.01299

**Published:** 2014-11-18

**Authors:** Friederike Range, Julia Jenikejew, Isabelle Schröder, Zsófia Virányi

**Affiliations:** ^1^Messerli Research Institute, University of Veterinary Medicine Vienna, Medical University of Vienna, University of ViennaVienna, Austria; ^2^Wolf Science CenterErnstbrunn, Austria; ^3^Department of Behavioural Biology, University of MünsterMünster, Germany

**Keywords:** quantity discrimination, domestic dogs, wolf, mental representation, domestication

## Abstract

Certain aspects of social life, such as engaging in intergroup conflicts, as well as challenges posed by the physical environment, may facilitate the evolution of quantity discrimination. In lack of excessive comparative data, one can only hypothesize about its evolutionary origins, but human-raised wolves performed well when they had to choose the larger of two sets of 1–4 food items that had been sequentially placed into two opaque cans. Since in such paradigms, the animals never see the entire content of either can, their decisions are thought to rely on mental representation of the two quantities rather than on some perceptual factors such as the overall volume or surface area of the two amounts. By equaling the time that it takes to enter each quantity into the cans or the number of items entered, one can further rule out the possibility that animals simply choose based on the amount of time needed to present the two quantities. While the wolves performed well even in such a control condition, dogs failed to choose the larger one of two invisible quantities in another study using a similar paradigm. Because this disparity could be explained by procedural differences, in the current study, we set out to test dogs that were raised and kept identically as the previously tested wolves using the same set-up and procedure. Our results confirm the former finding that dogs, in comparison to wolves, have inferior skills to represent quantities mentally. This seems to be in line with Frank’s (1980) hypothesis suggesting that domestication altered the information processing of dogs. However, as discussed, also alternative explanations may exist.

## INTRODUCTION

The ability to discriminate between different quantities is thought to be advantageous for any social species (Byrne, 1995) because it does not only help to guide foraging decisions in regard to different amounts of food (Normand et al., 2009) or the optimal quantity of preys (Panteleeva et al., 2012), but also to decide whether or not to engage in intergroup conflicts that are potentially risky. For example, using playback experiments, it has been shown that lions (*Panthera leo*; McComb et al., 1994), chimpanzees (*Pan troglodytes;* Wilson et al., 2001), male black howler monkeys (*Alouatta pigra;* Kitchen, 2004) and hyenas (*Crocuta crocuta*; Benson-Amram et al., 2011) are more likely to approach simulated intruders when facing favorable odds, i.e., in situations in which their own group outnumbers the intruder’s group. Similarly, an observational study on free-ranging domestic dogs (*Canis familiaris*) showed that dogs also adjust their behavior in intergroup conflicts to the number of opponents (Bonanni et al., 2011). In contrast to the above mentioned playback studies that suggest that animals may indeed make their decisions based on numerical information, observational studies, such as the one on free-ranging dogs, do not exclude the possibility that the animals rely on purely perceptual features. That is, they may rely on the overall amount, volume, or surface area covered by the animals in both groups because the rival as well as the own group are usually visible during intergroup encounters.

Experimental studies examining dogs’ abilities under more controlled conditions also suggest that dogs’ quantity discrimination skills may be limited in regard to relying on mental representations of various amounts. Ward and Smuts (2007), for instance, showed that dogs could discriminate between two small quantities of 1–5 items, if those were presented simultaneously and were visible the entire time. However, two dogs that were subsequently tested with quantities invisible during choice dropped substantially in performance, suggesting that the dogs might have relied on perceptual features during the visible discrimination rather than the provided quantity information. Somewhat contradictory, after manipulating the outcome of three simple calculations (1 + 1 = 2; 1 + 1 = 1; 1 + 1 = 3), West and Young (2002) found that dogs looked longer if the outcome violated their expectation than if not, suggesting that dogs did represent the (low) quantity of food items hidden sequentially. In a very recent study, however, dogs (*N* = 27) again failed to choose the larger of two quantities after a number of food items (0–4) was placed sequentially into two opaque cans (Macpherson and Roberts, 2013). This kind of sequential presentation with the food items invisible during the choice is advantageous compared to the simultaneous presentation since it requires the animals to modify their representation of each set’s content online as one item is added after the other. After all food items have been added to both sets, the animals have to then compare the two representations in order to choose the larger set without ever having seen the entire content of either set. This excludes the opportunity to choose based on perceptual features such as surface area or volume (see Beran, 2004 or Agrillo and Bisazza, 2014 for an extensive review on the various methodologies). Using this paradigm, all dogs excelled at differentiating 1 vs. 0, but were unable to successfully discriminate between any of the other presented ratios (Macpherson and Roberts, 2013).

Interestingly, wolves, the closest living relatives of dogs (Pang et al., 2009; Thalmann et al., 2013) performed remarkably well when tested in a similar paradigm (Utrata et al., 2012). In that study, we first trained 11 hand-raised wolves to choose four instead of a single food item in order to familiarize the animals with the experimental set-up. After the wolves reliably discriminated between invisible amounts of four and one food pieces, they were tested in all the other possible combinations of two sets of 1–4 items (1:2; 1:3; 2:3; 2:4; 3:4). In contrast to the dogs in Macpherson and Roberts’s (2013) study, the wolves were able to make quantitative judgments at all ratios.

These two canine studies are comparable to a certain degree, because both studies made sure that the animals needed to rely on the food quantities instead of choosing simply based on the amount of time needed to present the two sets of quantities. Obviously, inserting four pieces of food sequentially takes longer than dropping two pieces of food, which potentially allows the subjects to base their choices on temporal cues (Agrillo and Bisazza, 2014). Macpherson and Roberts (2013) prevented the dogs from doing so by equaling the amount of time used for inserting the different number of food items (that is presenting two pieces slower than four pieces). We used another method, and included neutral items (small pieces of stones) in additional control sessions so that the same number of items was inserted into the two cans (e.g., four food items in the left box and two food items + two stones in the right box; Utrata et al., 2012). The wolves still performed above chance in these conditions, suggesting that they base their decision on the quantity of food rather than any other perceptual features.

It is still possible, however, that the better performance of the wolves in our study compared to the dogs in the Macpherson and Roberts’s (2013) study is explained by the procedural difference that the wolves had received a training to familiarize them with the experimental set-up. Due to this training, the animals might have better understood what was required of them and they might have been more motivated and/or attentive to choose the larger quantity. Additionally, the wolves tested in our study might have had more experiences with cognitive experiments in general, which might potentially improve their performance.

If we can exclude these explanations, the difference found between dogs and wolves raises interesting evolutionary questions. Although addressing broader evolutionary issues is currently difficult because few studies on other canine species have been conducted (see Baker et al., 2011 for an example on coyotes), comparing only dogs and wolves may tell us about the effects of domestication. Frank (1980), for example, has suggested that the domestication process might have buffered dogs from the adaptive demands that favored higher cognition in wolves. Since many dogs do not need to search and hunt for food, defend their territories or find mating partners, natural selection might have relaxed on individual problem solving abilities and among them skills that require the use of mental representations of the physical environment. This hypothesis is in line with data showing that the relative brain size of dogs is smaller than that of wolves (Hemmer, 1990). If this hypothesis is correct, we may expect that dogs are less successful than wolves in quantity discrimination tasks that require the animals to base their choices on representations of different quantities. To test this question we need to compare dogs and wolves with comparable methods and after comparable raising histories.

Here, in order to test the representation-based abilities of dogs to discriminate different quantities and to compare them to wolves, we tested 13 dogs using the same experimental set-up that brought positive results in wolves. Importantly, since the 13 dogs have not only been trained and tested but also raised and kept under the same conditions as the previously tested wolves, we can assume that differences in socialization and previous experience cannot account for differences in the performance of the animals.

## MATERIALS AND METHODS

### ETHICAL STATEMENT

No special permission for use of animals (dogs) in such socio-cognitive studies is required in Austria (Tierversuchsgesetz 2012 – TVG 2012). The relevant committee that allows running research without special permissions regarding animals is: Tierversuchskommission am Bundesministerium für Wissenschaft und Forschung (Austria).

### SUBJECTS

The 13 dogs (*Canis familiaris*) participating in this study were born in different dog shelters in Hungary and were all crossbreeds (**Table 1**). They were separated from their mothers within the first 10 days after birth and were hand-raised and socialized by staff of the Wolf Science Center (WSC), Austria (for details of the raising methods please refer to Range and Virányi, 2014). At the time of this study, the dogs were living in four different packs in separate enclosures (2000 m^2^) at the WSC in Austria. The dogs were fed daily with dry food, and water was available *ad libitum*. Since puppyhood all animals had been trained regularly, several times a week and participated continuously in various cognitive and behavioral tests. They were rewarded with dry food, cheese or sausage. The training sessions, executed by professional animal trainers, consisted of obedience training and were conducted either in the test building or the testing enclosure in physical separation of the pack. The 11 wolves used for comparison have been raised and kept in the same manner as the dogs at the WSC (for details of the wolves see Utrata et al., 2012).

**Table 1:** Information on the subjects participating in this study.

Subject	Origin	Litter	Pack	Age	Sex	Participation			
						Training	Test	Time	Stone
Kilio	Paks, Hungary	1	1	3	M	x	x	x	x
Meru	Velence, Hungary	2	1	2.5	M	p.p.	n.p.	n.p.	n.p.
Nia	Paks, Hungary	3	1	1.5	F	x	x	x	x
Bashira	Paks, Hungary	4	1	2.5	F	p.p.	n.p.	n.p.	n.p.
Asali	Siofok, Hungary	5	2	2.5	M	x	x	x	x
Bora	Györ, Hungary	6	2	1.5	F	p.p.	n.p.	n.p.	n.p.
Nuru	Paks, Hungary	7	3	1.5	M	x	x	x	x
Layla	Györ, Hungary	6	3	1.5	F	p.p.	n.p.	n.p.	n.p.
Zuri	Paks, Hungary	7	3	1.5	F	x	x	x	x
Rafiki	Tengelic, Hungary	8	4	3	M	p.p.	n.p.	n.p.	n.p.
Hakima	Paks, Hungary	4	4	2.5	M	x	x	x	x
Maisha	Paks, Hungary	1	4	3	M	x	x	x	x
Binti	Siofok, Hungary	5	4	2.5	F	x	x	x	x

*M, male; F, female; x, animal participated in this phase of the study; p.p., participating partly; n.p., not participating*.

At the time of testing, the dogs and wolves were closely matched in age (average age dogs: 2.2 years; average age wolves: 2.6 years) and had comparable experience in regard to behavioral studies. All animals had participated in the same cognitive tests prior to this study ranging from personality to social learning tasks. Also, all animals participated in a quantity discrimination task when they were 1 year or younger, where they had to choose between 1 and 8 pieces of cheese put in front of them on two plates. That study aimed at testing the influence of a human on the choice of the animal (see Prato-Previde et al., 2008).

### EXPERIMENTAL SET-UP

**FIGURE 1:**
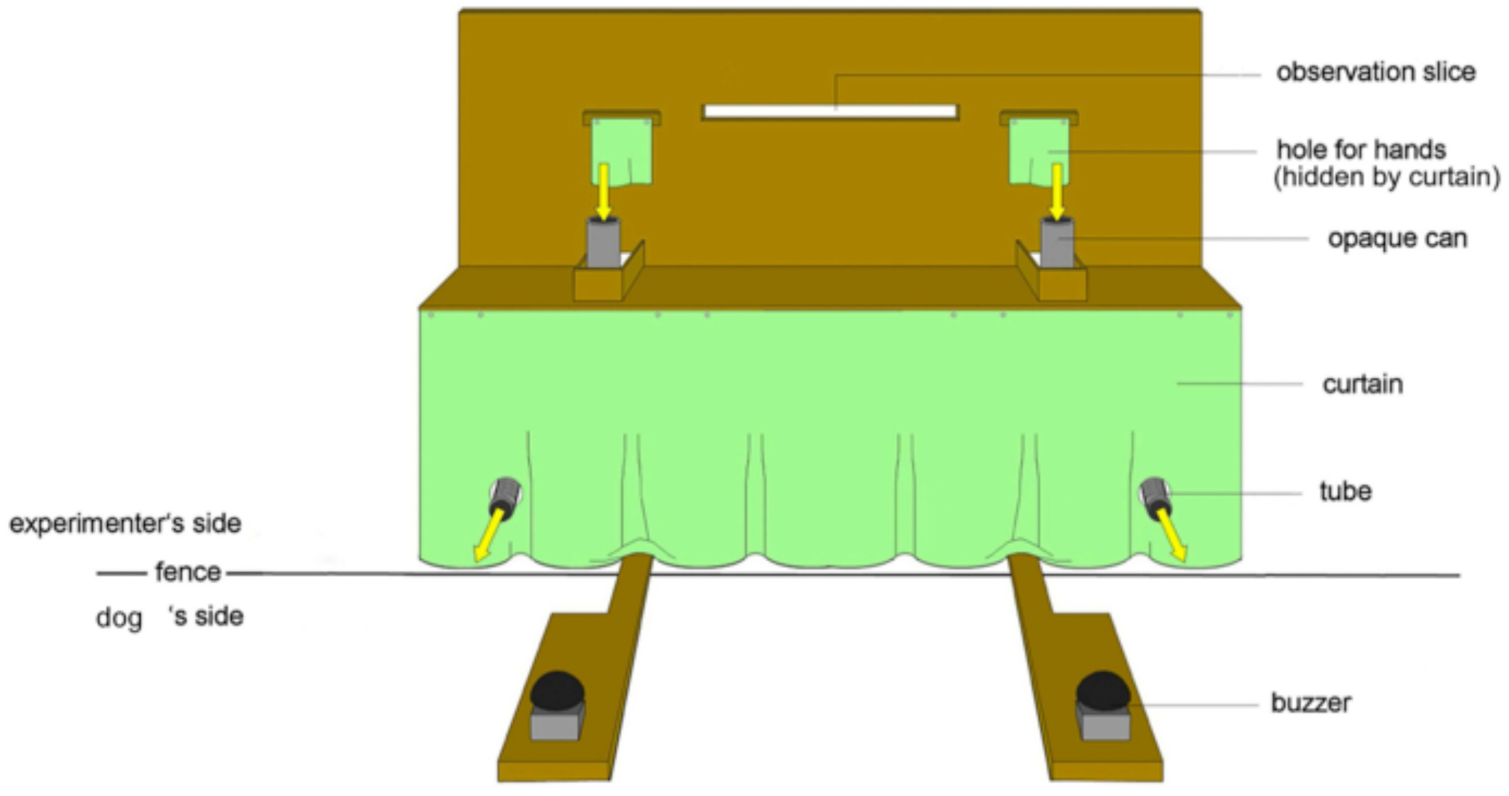
**The experimental set-up of the apparatus from the dog’s perspective.** The image shows the table, the two buzzers and the opaque cans with the rewarding tubes, which are leading from the air lock into the testing enclosure (from Utrata et al., 2012).

The experimental apparatus, consisting of a wooden table (170 cm × 40 cm × 60cm) with two opaque plastic cans (*h* = 14 cm, Ø = 8 cm) on the left and right side respectively, was placed directly next to the fence outside of the testing enclosure (**Figure 1**). The experimenter was sitting behind a visual barrier mounted on the top of the table and could see the table and the animal through an observation slit. The barrier had two holes for the experimenter’s hands directly above the cans to drop the food items directly into the cans. The cans were connected to plastic tubes, which led through the fence into the testing enclosure. To prevent the food items from sliding into the enclosure directly after insertion, the bottoms of the cans were closed by a wooden bar that could be removed by the experimenter. The visual barrier and the curtain below the table prevented the dogs from seeing the experimenter’s body in order to reduce a possible influence of unintentional cues. During the experiment the experimenter wore sunglasses, therefore the animals could not see her gaze through the observation slit. On the dogs’ side of the fence, a wooden panel was placed under each tube with a buzzer fixed on top.

### PROCEDURE

The experiment consisted of a training and a test phase (including two controls). The basic procedure was the same for all trials (except for training level 1): for each session the dogs were brought in a testing enclosure separated from the rest of the pack. The experiment began after the trainer had positioned herself about 1.5 m in front of the apparatus holding the dog on its collar. During the entire training and test procedures, the trainer had her eyes closed and her head held down so that she could see none of the experimenter’s actions. The experimenter prepared the required amount of food (or stones) and inserted both of her filled, closed hands into the holes above the opaque cans. Then she showed the dog one item at a time holding it visibly between two fingers while the rest of the food items were still hidden in her closed palm. Next, the experimenter called the dog’s name to get its attention and once it looked, the experimenter placed the item either onto the table in front of the can (*training steps 2 + 3 + 5*) or dropped it into the can (*training step 4, test and control trials*). After placing all items she had in her first hand, she showed her open palm to the dog. Then, she placed or inserted the items in her other hand on the table or into the second can in the same way. Whether she emptied her right or left hand first was pseudo-randomized within and across dogs, with the restrictions that she started on each side equally often and no more than three consecutive trials on the same side to avoid the development of a side preference.

After showing her second empty hand to the dog, the experimenter gave a signal to the trainer who released the dog giving a short command (“go”). Although in the training phases the animals had to step with their forepaw on the buzzer to provide an acoustic signal to clearly indicate their choice, in the testing phase choices were also counted if the dog used the buzzer without producing a signal, stepped on the wooden panel to which the buzzer was attached to, or touched the fence on the side of the can with its nose for at least 3 sec. This was implemented to avoid missing or misinterpreting the dog’s first choice by waiting too long for it to use solely the buzzer. If a correct choice was made, the experimenter released the items chosen by the subject by pulling out the plastic bar from under the can. If the items had been placed on the table (training steps 2, 3, and 5), the experimenter inserted them into the can from where they could slide into the enclosure. Incorrect choices were not rewarded except in training steps 3 and 4 (see below for details).

Three professional animal trainers were involved in testing the dogs. Moreover, two experimenters conducted the testing in the first five vs. the last 3 months. Since the experimenters were hidden behind the apparatus (see experimental set-up), we expected that this would not influence the performance of the animals. Nevertheless, we checked for a potential effect of experimenters by integrating them as a factor in the statistical analyses but we found no significant effect (*F*_1,5_ = 0.08, *p* = 0.8).

Yellow cheese pieces (Gouda, 1 cm × 1 cm × 1 cm) were used as reward and black stones of a comparable size were used in the control trials. Only one session per day was conducted with 1–2 days elapsing between sessions.

#### Training phase

During the five-step training phase, the animals were familiarized with the apparatus and the procedure. In *step 1*, using operant conditioning with a secondary reinforcer (clicker) and dry dog food as a reward, each dog was trained to press a buzzer reliably with its paw on command. No table was present and the rewarding was done by hand. First, only one single buzzer was available to train the dogs how to operate it with their paws. After successfully pushing the buzzer ten times in a row after a command was given, the second buzzer was introduced. The criterion to continue to step 2 was set at operating the buzzers 10 times in a row according to the side the trainer pointed at.

In *step 2*, the dogs were required to choose four pieces instead of one piece of cheese placed next to the opaque cans on the table in full view of the animals. The position of the two quantities and their order of placement were randomized and predetermined. Each session consisted of eight trials if the subject made no mistake. However, in case of a mistake, correction trials were administered, in which the combination of the previous trial was repeated until the animal chose the larger reward. The criterion to proceed to the next step was set at nine or more correct choices in the last 11 trials to assure that the animals made correct choices at least twice in each of the four possible combinations.

In *step 3*, the dogs had to discriminate between a visible piece of black stone and a visible piece of cheese placed on the table. Each session consisted of seven trials and criterion was set at six or more correct choices in a session.

*Step 4* equaled *step 3* with the difference that the piece of cheese and the stone were inserted into the opaque cans and were thus invisible during the choice. In *steps 3* and *4,* we always released the chosen item into the enclosure to allow the animals to inspect the stones in case they made the wrong choice. At the end of each trial, the trainer collected the stone and gave it back to the experimenter.

In *step 5*, similarly, to step 2, the dogs had to choose once again the larger of two visibly presented quantities (1 vs. 4 cheese pieces) to assure that they still chose the larger quantity after the other training steps. However, no correction trials were conducted and criterion was set at six or more correct choices in the last seven trials. The training procedure was identical to the one used with the wolves (Utrata et al., 2012).

#### Testing phase

***Quantity discrimination test***. In the testing phase, the animals were required to discriminate between two quantities (1 vs. 4, 1 vs. 3, 1 vs. 2, 2 vs. 4, 2 vs. 3, 3 vs. 4) that had been dropped one-by-one into the respective opaque cans and were therefore invisible during the choice. Randomizing the side and the placing order of the six conditions resulted in a total of 24 conditions. Each possible condition was repeated twice in a total of eight test sessions of six trials each.

***Time and stone control***. In order to investigate whether the dogs made their choices based on quantity discrimination or used alternative strategies, we conducted two control experiments. The aim of the first control experiment was to determine whether the animals actually compared the two quantities or, alternatively, if they solved the discrimination by either using the time interval it took to insert the different number of food pieces into the can or the total amount inserted on each side (“time/amount” *control*). In order to control for the difference in time and the total amount in each tube, we added stones to the smaller quantity of cheese pieces until both cans contained the same number of items as well as the same overall amount (stones had the same size as cheese).

However, since the stones were always added on the side with the fewer pieces of cheese, the animals could have easily chosen the bigger cheese amount in this control experiment by avoiding the sound the stones made when being dropped into the respective can. Therefore, in the second control experiment, we added an extra stone to both sides (e.g., 3 vs. 1: one can contained three pieces of cheese and one stone and the other can contained one piece of cheese and three stones).

Each control experiment consisted of four sessions of six trials each and tested only the following three (cheese) quantity pairs: 1 vs. 2, 1 vs. 4, 2 vs. 3. Accordingly, we had a pair with a small distance and a large ratio between sets (1 vs. 2), a pair containing a large distance and a small ratio between sets (1 vs. 4) and a pair with a large ratio and an intermediate distance (2 vs. 3).

### DATA ANALYSES

Initially we examined the performance of dogs in the training phase and compared them to the wolves (based on data from Utrata et al., 2012). We compared the number of trials the subjects needed to reach criterion for each level using two-tailed Mann–Whitney-*U*-tests.

In the test and control sessions, we analyzed whether non-quantity and quantity properties influenced the animals’ performance by calculating non-linear mixed effect (nlme) models using binomial distribution and including subjects and sessions as random factors. We tested the following non-quantity factors: (1) the side where the larger cheese quantity had been placed (‘side_larger quantity’), (2) the order of placing the two sets (larger amount placed first, ‘order_first’), (3) session (sessions 1–8, ‘sess’) and (4) we tested for the influence of the experimenter’s identity. In regard to the quantity factors we included the ratio of the two presented sets (‘ratio’: 0.25, 0.33, 0.5, 0.66, 0.75). Finally, species was included as last factor to test for differences between wolves and dogs. If the non-quantity factors proved to have no influence on the animals’ performance they were excluded from further analyses.

Furthermore, by comparing the data to chance level with one-sample *t*-tests, we tested for a general side bias and whether the number of trials in which the animals chose the bigger cheese amounts differed from chance level.

The data were analyzed using the statistical software *R* (version 2.15.2). Alpha was set at 0.05.

## RESULTS

### TRAINING PHASE

Eight of the 13 dogs that participated in this study (61.5 %) passed all training steps and were tested in the quantity discrimination test and control experiments (see **Table 2** for details). Of the five dogs that failed to complete the training, one did not learn to press the buzzer and four never reached the criterion of discriminating between 1 and 4 cheese pieces visibly presented (either in Step 2 or Step 5). In contrast, 10 of 11 wolves (90.9%) passed all training steps in the previous study participating in all subsequent test and control trials.

**Table 2:** Number of trials every subject needed to reach criterion and to enter the next step (step 2–5) in training phase.

Subject	Step 2 1 vs. 4 in full view	Step 3 Stone vs. cheese in full view	Step 4 Stone vs. cheese in can	Step 5 Same as step 2 without correction trials	Overall
Kilio	111	27	20	7	165
Meru	57	21	147	n.p.	
Nia	56	21	13	7	97
Bashira	n.p.				
Asali	86	35	7	7	135
Bora	111	42	126	n.p.	
Nuru	81	21	35	7	144
Layla	129	n.p.			
Zuri	85	49	21	8	163
Rafiki	126	n.p.			
Hakima	103	20	27	15	165
Maisha	99	14	49	23	185
Binti	72	28	6	7	113

*n.p., not participating (subject did not reach the criterion of the previous training level)*.

The remaining eight dogs did not differ from the wolves in the number of trials to reach criterion in training step 2, 4, and 5 (Mann–Whitney-*U* test: step2: *Z* = -1.07, *p* = 0.30; step4: *Z* = -1.70, *p* = 0.09; step5: *Z* = -1.17, *p* = 0.25). However, in step 3, where the animals had to discriminate between a piece of cheese and an equally sized black stone, the dogs needed more trials than the wolves to reach criterion (Mann–Whitney-U: *Z* = -2.672, *p* = 0.006).

### TESTING PHASE

#### Non-quantity factors

***Quantity discrimination test***. We found no significant impact of the side on which the larger food quantity was placed (NLME_side_larger_quantity_: *F*_1,843_ = 1.92, *p* = 0.17), the session (NLME_sess_: *F*_1,842_ = 0.23, *p* = 0.64) or whether the larger quantity was placed first (NLME_order_first_: *F*_1,850_ = 3.030, *p* = 0.080) on the dogs’ decision to choose the larger amount. Wolves did not differ from dogs in regard to the influence of these non-numerical factors (NLME_side_larger_quantity__×__species_: *F*_1,839_ = 0.65, *p* = 0.42; NLME_sess__×__species_: *F*_1,837_ = 0.06, *p* = 0.80; NLME_order_first__×__species_: *F*_1,846_ = 2.46, *p* = 0.12). Moreover, no side bias occurred either in the dogs or in the wolves (one-sample *t*-test: dogs: *t*_7_ = -0.12, *p* = 0.91; wolves: *t*_9_ = -0.64, *p* = 0.53).

#### Control conditions

Again, neither in the time nor in the stone control experiments was the dogs’ performance influenced by the session (NLME_sess_: time control: *F*_1,425_ = 0.01, *p* = 0.92; stone control: *F*_1,414_ = 0.93, *p* = 0.33). This lack of effect was independent of the species (NLME_sess.__×__species_: time control: *F*_1,421_ = 2.87, *p* = 0.09; stone control: *F*_1,410_ = 0.03, *p* = 0.87). Furthermore, no side bias was found in the two canines in either control (one-sample *t*-test: time control: dogs: *t*_7_ = 1.80, *p* = 0.12; wolves: *t*_9_ = -0.61, *p* = 0.56; stone control: dogs: *t*_7_ = 1.06, *p* = 0.33; wolves: *t*_9_ = 0.11, *p* = 0.91). However, while the placing order of the sets had no effect on the choices either dogs or wolves made in the time control experiments (NLME_order_: *F*_1,423_ = 0.06, *p* = 0.81; NLME_order__×__species_: *F*_1,420_ = 0.48, *p* = 0.49), in the stone control the wolves were more likely to choose the larger food amount when it was placed second in contrast to the dogs whose choice was not influenced by the placing order (NLME_order_: wolves: *F*_1,219_ = 7.530, *p* = 0.007; dogs: *F*_1,190_ < 0.01, *p* > 0.99).

#### Quantity factors

**FIGURE 2:**
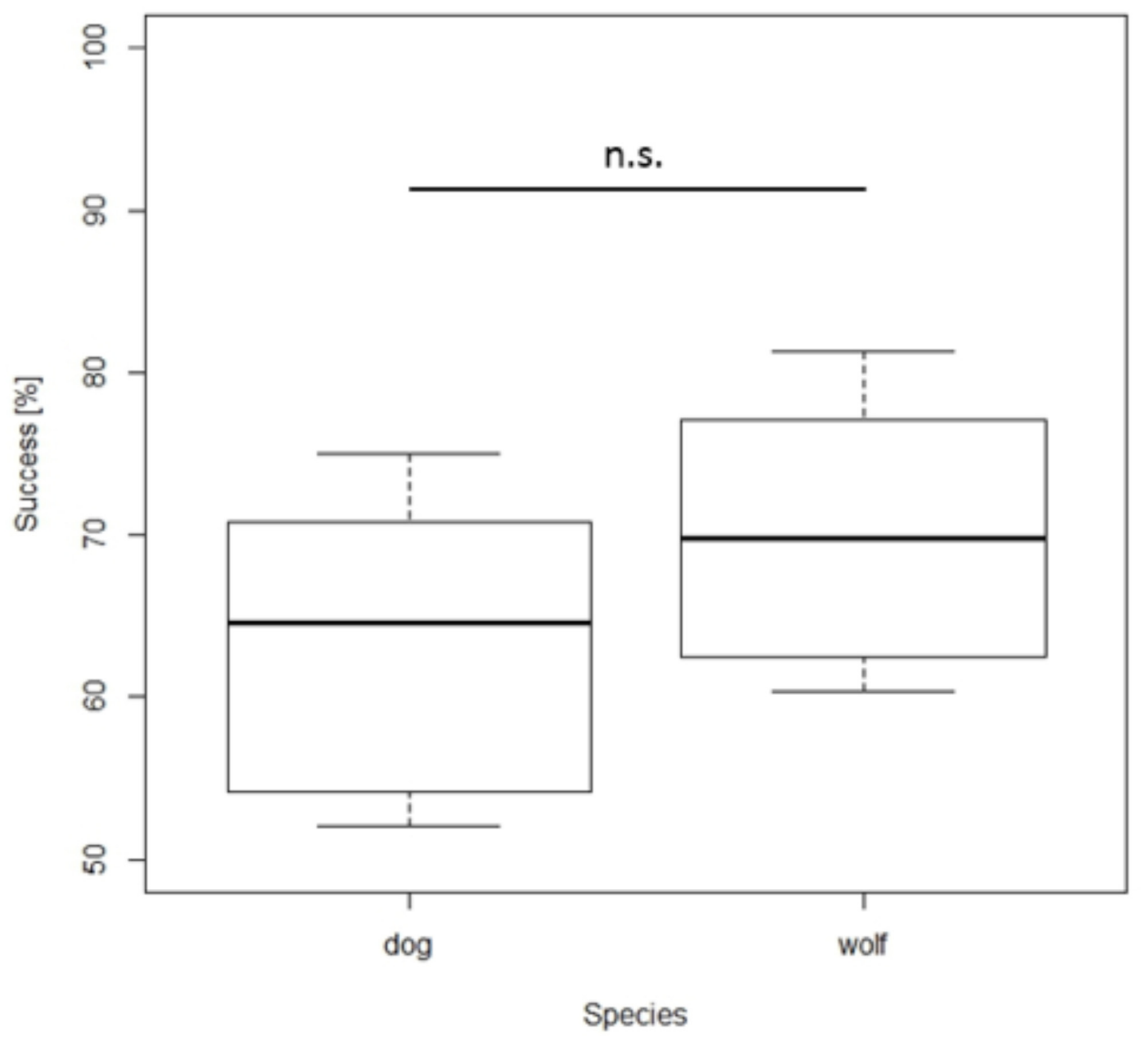
**Box plots showing the percentage of total number of correct choices during the quantity discrimination test.** Boxes represent the interquartile range, bars within boxes are median values, whiskers indicate the 5th and 95th percentiles. n.s. = non-significant.

Quantity discrimination test. Overall, dogs and wolves chose the larger quantity significantly above chance (one sample *t-*test: dog: *t*_7_ = 4.219, *p* = 0.004; wolf: *t*_9_ = 8.881, *p* < 0.001). Although wolves performed slightly better than dogs, choosing the larger amount in 70.42% of the trials compared to 63.28% in the dogs, this difference was not significant (NLME_larger_quantity_: *F*_1,16_ = 3.380, *p* = 0.080, **Figure 2**). The same results were found when excluding the combination 1 vs. 4, the pair known from the training phase: Dogs and wolves chose the larger quantity more often *(one sample t-*test – dog: *t*_7_ = 3.742, *p* = 0.007; wolf: *t*_9_ = 8.249, *p* < 0.001) and did not differ significantly from each other (NLME_larger_quantity_: *F*_1,16_ = 2.64, *p* = 0.12).

However, when looking at each numerical pair separately, it turns out that, while wolves were significantly above chance at all tested ratios, dogs performed above chance level only at ratios at or below 0.5 (see **Table 3**).

**Table 3:** Performance of dogs and wolves according to the ratios tested in the quantity discrimination test.

		Quantity discrimination test				Time control				Stone control			
Ratio	Sets	Dog		Wolf		Dog		Wolf		Dog		Wolf	
0.25	1:4	*t*_7_ = 2.58	*p* = 0.036	*t*_9_ = 5.28	*p* = 0.001	*t*_7_ = 3.33	*p* = 0.013	*t*_9_ = 3.43	*p* = 0.008	*t*_7_ = 0.80	*p* = 0.45	t_9_ = 3.32	p = 0.009
		67.19%		77.50%		71.88%		71.25%		53.13%		69.74%	
0.33	1:3	*t*_ 7_ = 4.00	textitp = 0.005	*t*_9_ = 5.10	*p* = 0.001								
		75.00%		71.60%									
0.50	1:2	*t*_7_ = 4.78	*p* = 0.002	*t*_9_ = 6.10	*p* < 0.001	*t*_7_ = -2.50	*p* = 0.041	*t*_9_ = 2.64	*p* = 0.027	*t*_7_ = 1.16	*p* = 0.29	*t*_9_ = 2.10	*p* = 0.065
		71.88%		73.42%		39.06%		65.82%		54.69%		60.53%	
0.50	2:4	*t*_ 7_ = 3.99	*p* = 0.005	*t*_9_ = 4.20	*p* = 0.002								
		75.00%		68.75%									
0.67	2:3	*t*_ 7_ = 0.61	*p* = 0.56	*t*_9_ = 3.21	*p* = 0.011	t_7_ = -0.36	*p* = 0.73	*t*_9_ = 2.95	*p* = 0.016	*t*_7_ = 0.24	*p* = 0.82	*t*_9_ = 3.798	*p* = 0.004
		53.13%		62.96%		48.44%		62.96%		51.56%		71.05%	
0.75	3:4	*t*_ 7_ = -0.68	*p* = 0.52	t_9_ = 3.82	*p* = 0.004								
		46.88%		68.35%									

*The *p*-values indicate whether the subjects performed above (light gray cells) or below (dark gray cell) chance level (one-sample *t*-tests). Stone control: wolves: 67.11%; dogs: 53.13%*.

**FIGURE 3:**
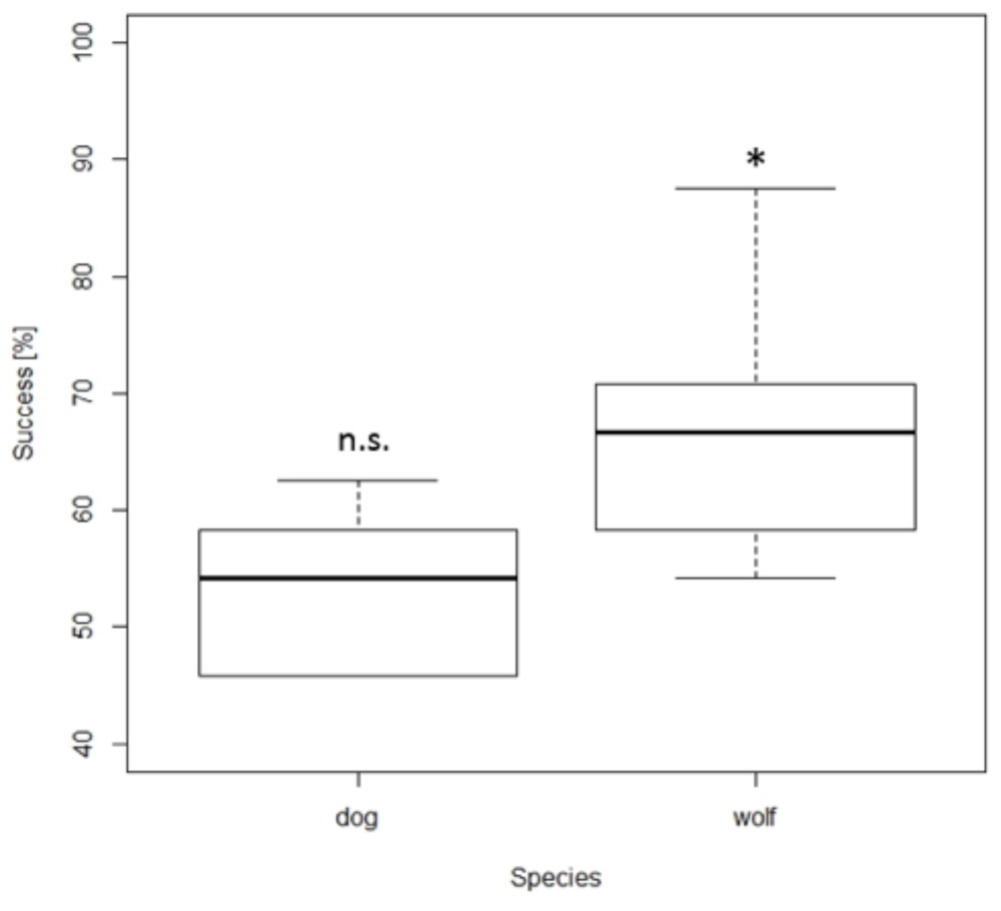
**Box plots showing the percentage of total number of correct choices during the time control.** Boxes represent the interquartile range, bars within boxes are median values, whiskers indicate the 5th and 95th percentiles. ^∗^ *p* < 0.05.

***Time control***. Importantly, while the wolves selected the larger amount above chance level in the time control experiment (67%; one sample *t-*test: *t*_9_ = 5.164, *p* = 0.001), dogs’ performance did not differ from chance level (53%; *t*_7_ = 1.27, *p* = 0.24, **Figure 3**). Furthermore, the wolves’ performance was not influenced by the ratio between the presented sets whereas the dogs’ performance improved with lower ratios (NLME_ratio_: dog: *F*_1,878_ = 11.84, *p* < 0.001, wolf: *F*_1,243_ = 1.32, *p* = 0.30). More precisely, the dogs’ performance was above chance with a ratio of 0.25, below chance with a ratio of 0.5, and at chance level with a ratio of 0.67 (**Table 3**). The wolves performed above chance at all three ratios (**Table 3**).

***Stone control***. In the stone control, the wolves again chose the larger quantity more often than the dogs (NLME_larger_quantity_: *F*_1,418_ = 8.680, *p* = 0.003), performing above chance level while the dogs performed at chance level (one sample *t-*test*:* wolfs: 67%; *t*_9_ = 4.391, *p* = 0.002; dogs: 53 %; *t*_7_ = 1.34, *p* = 0.22). Ratio had no influence on the canines’ performance (NLME_ratio_: *F*_1,415_ = 0.03, *p* = 0.86; NLME_ratio__×__species_: *F*_1,412_ = 0.02, *p* = 0.90). Again looking at the separate ratios, while dogs performed at chance level at all three ratios, wolves performed above chance level at 0.25 and 0.67, but only approached significance at 0.5 (**Table 3**).

## DISCUSSION

Overall, we found that the dogs as well as the wolves chose the larger quantity above chance level in the quantity discrimination test. However, a closer look at the performance of the subjects according to the tested ratios revealed that while wolves performed above chance even with high ratios (e.g., 0.67 and 0.75), in these conditions dogs performed at chance level.

Our dog results are in line with previous studies on canines suggesting that at least dogs and coyotes have problems discriminating between two small quantities of high ratios (0.75 and 0.66) if these are invisible at the moment of the choice (Ward and Smuts, 2007; Baker et al., 2011; Macpherson and Roberts, 2013). Although our dogs performed above chance in the initial quantity discrimination task up to a ratio of 0.5, their performance dropped if we controlled for the total amount/time to insert the items. The same failure of dogs in quantity discriminations of up to four items was observed by Macpherson and Roberts (2013), who also controlled for the time it took to insert the items. Our results are important since they exclude the potential other explanations that Macpherson and Roberts (2013) offered for the dogs’ poor performance: first, they argued that the fact that the dogs received a food reinforcement even if they chose the smaller amount might have confused them. During normal obedience training, dogs usually only receive rewards for correct responses and thus they might not have understood that they were required to choose the larger reward. Due to the training in our experimental set-up, our dogs learned the rule to choose the larger of two presented sets and only were tested once they had reached a certain criterion rendering this explanation for their poor performance unlikely (see also Agrillo and Bisazza, 2014 for a discussion on training on quantity discrimination skills). This conclusion is also supported by the positive results of the initial discrimination task, where the dogs were able to discriminate between the two quantities up to a ratio of 0.5. Second, Macpherson and Roberts (2013) point out that the presence of the human experimenter could have made it very difficult for the dogs to engage in a cognitive task. However, since we did not test pet dogs, but dogs that live in packs and that are, on top of that, accustomed to being exposed to problem solving tasks on a daily basis, we can also rule out this alternative explanation for the dog’s failure. Accordingly, our results confirm the results by Macpherson and Roberts (2013), and the two studies provide rather firm evidence that dogs have limited capabilities to represent the number of food items in the two containers and to mentally compare the two quantities.

Interestingly, in contrast to dogs, wolves were able to discriminate between high ratios of small quantities when tested in the same task (Utrata et al., 2012). There are several explanations that could potentially explain the better performance of the wolves:

First, canines have an extraordinary sense of smell compared to humans, which could theoretically allow them to discriminate at least quantities of lower ratios based on olfaction. However, there is little indication that canines would rely on their olfactory cues in such a situation if not specifically trained to do so. For example, dogs do not rely on their sense of smell in two choice tasks if visual information is provided by a human experimenter (Szetei et al., 2003), which we also confirmed for our wolves (unpublished data). Moreover, while the popular consensus is that olfaction is very important for hunting (Asa and Mech, 1995), two studies that experimentally investigated the role of olfactory, auditory and visual cues found that visual cues are the most important ones for hunting in red foxes (Österholm, 1964) and coyotes (Wells, 1978; Wells and Lehner, 1978). Moreover, in a study comparing the performance of our wolves and dogs in a local enhancement task, we found that, while both species primarily relied on visual information, the dogs actually used their sense of smell more than the wolves (Range and Virányi, 2013). These results might suggest that in canines visual information may easily override odor cues if searching for hidden food items (but see Gazit and Terkel, 2003 for a contrary example in explosives detection trained dogs). Finally, during our experiment, we never cleaned the tubes between trials or animals which likely lead to an overall strong cheesy smell of both tubes [probably similar to the olfactory control used by Macpherson and Roberts (2013)] making it potentially very difficult for the canines to discriminate between quantities that differed only by one – as the wolves were able to do.

Second, the observed difference between wolves and dogs could be due to dogs having worse eyesight than wolves (Miller and Murphy, 1995), making it difficult for them to discriminate between the single food items or discriminate between stones and cheese. The dogs’ performances in training step 3, where they needed more trials to successfully discriminate between cheese and stones than wolves may support this claim. However, the dogs did reach the criterion in the training suggesting that they learned to successfully discriminate between the food items and stones. Moreover, they could solve the sets with the small ratios in the quantity discrimination task and time control, suggesting that their eyesight was not the limiting factor either.

Yet another explanation could be that the dogs were less motivated than the wolves to solve the task. However, the dogs did solve several combinations in the quantity discrimination test and in the lowest ratio of the first control condition. Moreover, since both wolves and dogs were reared and kept under similar conditions, differences in socialization or reinforcement, that have been shown to influence performance in problem solving skills (Frank, 2011) can be excluded as influencing factors in our set-up.

Finally, it is possible that due to domestication, dogs possess a weaker ability to mentally represent and discriminate quantities in comparison to wolves. More specifically, Frank (1980) suggested that domestication has changed dogs’ information processing (including internal representations) in comparison to their closest wild-living relative, the wolf (Pang et al., 2009; Thalmann et al., 2013) due to the buffering effect of humans (see Introduction). The fact that the dogs are less successful than wolves in the quantity discrimination task when tested with the same experimental procedure is in line with this hypothesis.

The observed differences between wolves and dogs in our task are not unusual. Differences in performances between closely related species have also been reported for elephants for instance. African elephants are affected by the numerical ratio (Perdue et al., 2012), while Asian elephants seem to be insensitive to it (Irie-Sugimoto et al., 2009). Nevertheless, based on currently available data, it would be difficult to make broader arguments about evolutionary origins (e.g., social vs. solitary species) of quantity discrimination skills relying on mental representations. To date, while several animal species such as horses (Uller and Lewis, 2009), robins (Garland et al., 2012), rhesus macaques (*Macaca mulatta;* Hauser et al., 2000; Sulkowski and Hauser, 2001), and chimpanzees (*Pan troglodytes*; (Beran, 2001, 2004; Hanus and Call, 2007) have been shown to successfully discriminate between quantities using an item-by-item procedure, only very few studies controlled for the time component, making direct comparisons of the performance between species difficult. For example, although robins successfully differentiate groups differing by one unit up to 7 vs. 8 (Garland et al., 2012), great apes up to 5 vs. 6 (Hanus and Call, 2007) or 3 vs. 4 (Beran, 2004), macaques up to 3 vs. 4 items (Hauser et al., 2000; Sulkowski and Hauser, 2001) and horses only up to 2 vs. 3 items (Uller and Lewis, 2009), only the study on horses (Uller and Lewis, 2009), one on macaques (Sulkowski and Hauser, 2001) and one on chimpanzees (Beran, 2004) properly controlled for the time it took to insert the food items.

In conclusion, our study supports results by Macpherson and Roberts (2013) showing that dogs seem to differ from several other animals species including their closest living relative, the wolf, in that they are unable to mentally represent quantities and discriminate between them if other potential cues are controlled for. Further studies with domesticated and non-domesticated species characterized by different feeding ecology or social organizations have to show whether a different use of mental representations altered by domestication is responsible for this as proposed by Frank (1980), or whether other ecological differences parallel this cognitive difference in dogs and wolves as well as in other species.

## Conflict of Interest Statement

The authors declare that the research was conducted in the absence of any commercial or financial relationships that could be construed as a potential conflict of interest.
